# The Liminal Leisure of Disadvantaged Young People in the UK Before and During the COVID-19 Pandemic

**DOI:** 10.1007/s43151-021-00064-2

**Published:** 2021-12-06

**Authors:** Nicholas Woodrow, Karenza Moore

**Affiliations:** 1 School of Health and Related Research, University of Sheffield, Regent Court, S1 4DA, Sheffield, England; 2 School of Geography, Politics and Sociology, Newcastle University, King’s Gate, NEI 7RU, Newcastle upon Tyne, England

**Keywords:** Young people, Liminal leisure, Lockdown, COVID-19

## Abstract

The global COVID-19 pandemic has created, exposed and exacerbated inequalities and differences around access to—and experiences and representations of—the physical and virtual spaces of young people’s leisure cultures and practices. Drawing on longstanding themes of continuity and change in youth leisure scholarship, this paper contributes to our understandings of ‘liminal leisure’ as experienced by some young people in the UK before and during the COVID-19 pandemic. To do this, we place primary pre-pandemic research on disadvantaged young people’s leisure spaces and practices in dialogue with secondary data on lockdown and post-lockdown leisure. Subsequently, we argue that existing and emergent forms of youth ‘leisure liminality’ are best understood through the lens of intersectional disadvantages. Specifically, pre-existing intersectional disadvantages are being compounded by disruptions to youth leisure, as the upheaval of the pandemic continues to be differentially experienced. To understand this process, we deploy the concept of liminal leisure spaces used by Swaine et al Leisure Studies 37:4,440-451, (2018) in their ethnography of Khat-chewing among young British Somali urban youth ‘on the margins’. Similarly, our focus is on young people’s management and negotiation of substance use ‘risks’, harms and pleasures when in ‘private-in-public’ leisure spaces. We note that the UK government responses to the pandemic, such as national and regional lockdowns, meant that the leisure liminality of disadvantaged young people pre-pandemic became the experience of young people more generally, with for example the closure of night-time economies (NTEs). Yet despite some temporary convergence, intersectionally disadvantaged young people ‘at leisure’ have been subject to a particularly problematic confluence of criminalisation, exclusion and stigmatisation in COVID-19 times, which will most likely continue into the post-pandemic future.

## Introduction

Despite the COVID-19 pandemic being described as a ‘great leveller’ (Jones 2020), there is emerging evidence that its differentiated negative impacts reflect and exacerbate underlying inequalities (Marmot and Allen 2020) and re-establish exclusionary social hierarchies (Pfaller 2020). The COVID-19 pandemic has exposed the disadvantaged positions from which many young people must negotiate not only their education and employment transitions, but also their participation in leisure (The Audience Agency 2021). Leisure participation exclusion is being exacerbated by the intersecting crises of the COVID-19 pandemic, chronic underinvestment in state provision and related services and access to safe play and leisure facilities (Children’s Commissioner 2020a, b). The response to the COVID-19 pandemic in the UK and beyond (Han et al. 2020) has seen national and regional lockdowns restricting people’s activities and movements through stay-at-home instructions enforced through emergency legal powers (Greene 2020). In spring 2020, nearly all UK leisure spaces outside the home closed, with opportunities for leisure, or rather leisure involving physical proximity, essentially stopped. Paradoxically, despite leisure spaces closing, available leisure time increased for some (Bond et al. 2020). Leisure plays an ‘essential’ role in young people’s lives and wellbeing (Lashua et al. 2020; Roberts 2011). Evidence is now emerging of the largely negative impact of restrictions and lockdown leisure upon young people’s wellbeing (Roberts 2020; NHS Digital 2020). Those with existing mental health conditions have been particularly affected (YoungMinds 2020a). Worryingly this is set against the backdrop of a perpetual crisis in the NHS’s Child and Adolescent Mental Health Services (CAMHS) capacity and provision (Children’s Commissioner 2020b), suggesting increased challenges for those working with young people in already stretched services. Lockdown restrictions, socioeconomic conditions and future uncertainties have contributed to feelings of anxiety, loneliness and isolation in the context of a loss of coping mechanisms, most notably social support outside the family ‘unit’ (YoungMinds 2020b; Lisitsa et al. 2020). Such conditions may have detrimental long-term impacts for young people’s health and wellbeing (Orben et al. 2020).

During the pandemic, young people have been ordered to ‘stay at home’ to stay safe (Cabinet Office 2020). However, this ignores how access to safe and secure home spaces is profoundly shaped by socioeconomic disadvantage and inequality (Rosenthal et al. 2020). Indeed, some young people are expected to navigate living, working and/or studying from home in cramped, unsuitable, and unsafe housing (Leavey et al. 2020; Rosenthal et al. 2020). Similarly, the removal of physical ‘safe spaces’ of leisure and social interaction such as youth clubs, school/college/university or simply the streets, alongside inequitable access to virtual technologies and spaces (Honeyman et al. 2020), has proved particularly problematic for some young people (LGBT Foundation 2020), although invaluable for others (Hanckel and Chandra 2021). Through differentiated experiences of lockdown, including inequitable efforts to monitor and control youth ‘at leisure’ (Gabriel et al. 2021), the COVID-19 pandemic has spotlighted uncomfortable and neglected truths about inequalities, namely, that not all young people have unlimited access to technologies and digital forms of social interaction and that not all young people have safe spaces at home or even safe homes.

When the UK entered lockdown enforced by legislation on 23rd March 2020, any social gathering indoors and outdoors became ambiguous and ‘suspect’. COVID-19 measures have been in place in some form across the UK between March 2020 to November 2021, with oscillations between strict lockdown measures and partial reopening of licensed leisure spaces, dependent ostensibly on COVID-19 rates in geographical locations. The police were granted a variety of powers to punish lockdown infringements such as protests, demonstrations, vigils, socialising in streets, parks, house parties, free parties, illegal raves and illegal pay parties. During lockdown in England and Wales, police could dispense a Fixed Penalty Notice (FPN) to those deemed to be breaching ‘the rules’, starting at £200, rising to £6,400, with fines up to £10,000 for large gatherings or parties (Cabinet Office 2020). In January 2021, the UK government announced new fines for COVID-19 house parties (of more than 15 people) starting at £800 (BBC News 2021). As the COVID-19 pandemic continues, albeit in a changed form given the UK vaccine programme (November 2021), we have seen continued disruption to licit and illicit youth leisure spaces and practices, including licensed venue closures, vaccine passport legislation and further anti-rave legislation. Some leisure venues such as nightclubs in Northern Ireland have been shut for a 20-month period (Ross 2021). However, exclusion does not eradicate the desire for leisure, nor does the regulation of space prevent leisure practices, including substance use. Despite associated health and enforcement risks, young people continued to socialise in lockdown (Lashua et al. 2020; Roberts 2020). Young people’s differentiated responses to lockdown measures which were framed by the UK press as irresponsible and reckless (BBC News 2020) may be less ‘rebellion’ and more ‘reaction’ to continued conditions of exclusion, social isolation and loneliness (YoungMinds 2020a, 2020b). For some young people, ‘safe’ leisure remains unavailable, and available leisure possesses new ‘risks’ to be managed.

Understanding what the COVID-19 pandemic means for disadvantaged young people’s leisure presents a considerable challenge. However, youth studies scholarship has long been concerned with what it means to be young and disadvantaged (MacDonald and Marsh 2005; MacDonald et al. 2020). Young people’s experiences in the *pre*-pandemic era therefore help us understand manifestations of disadvantage in COVID-19 times and beyond. This is how we might avoid the ‘covidisation’ (a single lens) of research, without ignoring the intersections of the COVID-19 pandemic with other crises in youth transitions and cultures. To foreground continuity and change, we combine findings from a pre-pandemic study exploring disadvantaged young people’s risk perceptions and practices (Woodrow 2017), with more recent ethnographic observations of youth leisure practices as well as media representations of youth ‘at leisure’ during the pandemic (March 2020 to May 2021). The latter are part of an ongoing longitudinal mixed methods study of young people’s illicit drug use practices in a range of public and private/domestic spaces by Moore. In so doing, we place primary pre-pandemic research around disadvantaged young people’s leisure spaces and practices in dialogue with secondary data on lockdown leisure. On these combined foundations, we offer our thoughts on enduring and emergent ‘risks’, harms and pleasures of leisure spaces and related practices. At this juncture, we note that psychoactive substance use is a particularly important focus especially when illicit—as with street drinking or cannabis use—as young people may retreat to liminal leisure spaces to ‘stay safe’ from ‘risks’ such as police attention. We discuss how some young people have been portrayed and treated as ‘risky’, how they have been policed and how they manage and negotiate their leisure and substance use practices before and during COVID-19 times.

## Intersectional Perspectives on Youth Leisure

The past few decades have seen a shift in how inequalities are theorised, with an appreciation of the interplay or intersections between the privileges and disadvantages, and opportunities and constraints, apparent in non-essentialist inclusionary models of ‘identity’ (Collins and Bilge 2020; Hill 2015). Intersectionality was first deployed as a metaphor and developed into a powerful analytical concept by the Black legal feminist scholar Kimberle Williams Crenshaw (1989, 1991). Intersectionality seeks to explore and understand the effects of systems of inequality upon the most marginalised (Collins and Bilge 2020) and to look beyond notions of the individual as being subject to (dis)advantage in an ‘additive’ way. Intersectionality may be used then to appreciate interactions between (dis)advantages, whilst avoiding deploying it ‘as a theory of double or multiple oppression based on a positivist approach to categories (e.g., race, ethnicity, gender, gender identity, sexuality, disability, age, citizenship)’ (Carastathis 2016:4). Instead of an additive or multiplicity approach to disadvantage, we use intersectionality as a provisional concept (Crenshaw 1991; Carastathis 2014) to ‘think about how we think’ (Carastathis 2016:4) about how some young people ‘at leisure’ are produced as problematic social categories by those with the power to define, dominate and control (Blackman and Rogers 2017; Gabriel et al. 2021). This enables an exploration of how young people’s leisure and related practices such as substance use are shaped by intersectional disadvantages and how in turn intersecting crises may (re)produce further ‘risks’ and harms to those already experiencing these disadvantages, ‘risks’ which must be negotiated and managed by the young people in situ. An example of how the COVID-19 pandemic has intensified existing disadvantages is the exacerbated risk of involvement in the criminal justice system resulting from the differential policing of poor black urban communities around both illegal drugs and COVID-19 laws (House of Commons/House of Lords 2021). Intersectionality as a provisional concept can help explore the complexity and diversity of young people’s leisure experiences, especially in COVID-19 times, and help explain how these experiences are shaped by relational positions of intersectional (dis)advantage and marginalisation.

Echoing wider debates around the cultural and structural influences upon the lives of young people (MacDonald et al. 2020), disadvantaged young people have long been excluded from various leisure spaces, for example, the commercialised licensed venues of night-time economies (NTE). Intersectional disadvantages produce systematic patterns of inclusion and exclusion, shaping young people’s leisure participation (Wilkinson 2015). At the height of the COVID-19 pandemic, youth leisure exclusion was experienced more broadly by young people. Indeed, control measures and restrictions on legal licensed NTE leisure spaces in the UK such as night-time curfews and the closure of all pubs, bars, restaurants and nightclubs (Coronavirus Act 2020), as well as on unsupervised leisure spaces used by young people—such as private domestic spaces, flat and house parties (Ravn and Duff 2015), and public spaces, streets and parks (Robinson 2009)—resulted in ever more intense public, media and police scrutiny. In this, those experiencing leisure exclusion based on intersectional disadvantages have been particularly vulnerable to the criminalisation of social interaction in public and semi-public spaces (Brown 2013) and the proactive policing of drugs before and during the UK lockdown (Measham and Moore 2008; Metropolitan Police 2020). This is supported by a House of Commons/House of Lords (2021) report which concludes the way in which Fixed Penalty Notices (FPNs) have been used during the pandemic disproportionately penalises certain groups according to age, gender, race/ethnicity and social deprivation (see below for further discussion). A key continuity here is the historical positioning of some young people as simultaneously ‘at risk’ and ‘risky’, particularly those from racialised minority groups. A key change has been the UK government attempts to limit—and criminalise—much social interaction for a public health good and the negative implications of this criminalisation for young people. In COVID-19 times, whilst everyone is ‘at risk’ and ‘risky’, young people are represented as riskier than others, especially when ‘at leisure’, with this negative positionality commonly attributed to our most intersectionally disadvantaged young people.

## Continuity and Change: the Pre-pandemic North of England Town Study of Disadvantaged Young People ‘at Leisure’

The pre-pandemic study this paper draws upon explored the leisure practices and substance use, as well wider negotiation of employment transitions, of a sample of socioeconomically disadvantaged young people in a Northern Town in England (Woodrow 2017). Falling in the lowest quintile of the 2019 English indices of multiple deprivation, and similar to post-industrial areas nationally and internationally (O’Gorman 2016), the data collection site had a range of interconnected deprivations including: poverty; high levels of youth unemployment; poor educational attainment; high crime rates; and poor health profiles. Data collection took place between April 2015 and January 2016 and included observations in participants’ public leisure spaces, in-depth interviews and short anonymous surveys with young people aged between 14 and 24. The pre-pandemic study produced 24 in depth interviews with 27 young people lasting between 20 and 80 min. Data was transcribed and analysed using thematic analysis. Ninety-two percent of the sample was White British male, with many completing education or beginning to seek employment. Over 90% were living at their parental/family homes (Woodrow 2017).

Young people were recruited for participation in the pre-pandemic study during youth service outreach work sessions in public leisure spaces, with face-to-face interviews conducted in local public spaces such as cafes. Despite initial enthusiasm for participation, it proved challenging to secure interviews with young people, with many not attending arranged meetings, as due to the level of disadvantage, many participants did not have mobile phones or credit to allow meetings to be confirmed or altered. The challenges of engaging and accessing young people have been further exacerbated through COVID-19 restrictions, as some young people became close to ‘unreachable’ to youth researchers and youth workers (see below for further discussion). In the pre-pandemic study, to ensure that the perspectives and experiences of such young people were not missed, data was also collected through observations and recorded informal conversations in public leisure spaces. Surveys were also undertaken as in situ structured interviews, enabling conversation between the researcher and participant(s) during completion. This provided rich data, and the opportunity to observe interactions between young people in their own leisure spaces, again much missed by youth researchers and outreach workers during the pandemic.

## Risk Perceptions of the ‘Risky’ and ‘at Risk’: Then and Now

The North of England town study explored disadvantaged young people’s risk perceptions around substance use in mainly street-based leisure spaces and times (Woodrow 2017). The concept of ‘risk’ has become a central feature of contemporary society and has been used to frame all aspects of young people’s lives, including leisure spaces and practices (Bengtsson and Ravn 2018). It is crucial to reflect on the concept of ‘risk’ to engage critically with this framing. ‘Risk’ has typically been understood through a rational actor model, where ‘risky practices’ are seen to be undertaken by young people due to naive lack of awareness or misunderstandings of associated issues (Mason et al. 2013). This rationalist framework has informed dominant understandings of young people’s leisure practices including substance use but has been widely critiqued (Tulloch and Lupton 2003). In contrast, a sociocultural risk framework sees ‘risk’ as being embedded in social and cultural contexts (Pilkington 2007). This perspective foregrounds social and cultural contexts and meanings to better understand young people’s engagement with risks, harms and pleasures and ‘risky practices’ (Graham et al. 2018). Further, recent developments in intersectional risk theory (Nygren et al. 2020) drawing on feminist and Foucauldian understandings of gendered risks (Hannah-Moffat and O’Malley 2007) helps youth scholars identify ways in which ‘risk’ governance and regulation regimes such as drug prohibition (re)produce inequalities among young people.

The North of England town study participants conceptualised risks, harms and pleasures of their leisure and substance use practices—as well as their engagement with authority—primarily as being personal, immediate, acute and tangible, rather than abstract, potential, future-situated, chronic and long-term. Practices that were not perceived to be associated with such ‘immediate’ issues were differentiated as less risky, irrespective of their potential for long-term harm, tobacco consumption being one clear example. The participants held potentially erroneous beliefs around their abilities to avoid negative and long-term harms such as criminalisation (discussed below), with such beliefs shaping their current and future leisure practices. How and where our participants located ‘risk’ for example within a specific substance, practice and/or place, how they perceived associated pleasures and how they accessed and engaged with ‘risk media’ (lay and expert knowledge) were crucial to its negotiation. We suggest that applying this conceptualisation to young people’s leisure practices more generally in COVID-19 times can help us understand young people’s heterogeneous responses to lockdown and later post-pandemic leisure landscapes as nuanced, relational and shaped by intersectional disadvantages. Emerging research for example suggests that young males took more health ‘risks’ during lockdowns and were more likely to break lockdown rules than young women (Smith et al. 2020), with ‘risk’ beliefs around lower susceptibility to catching COVID-19 and spreading the virus shaping this (Levita 2020). Appreciating young people’s conceptualisations of ‘risks’ as being largely focused on the immediate (now) rather than potential (in ‘the future’) helps explain some young people’s adherence or otherwise to social distancing and lockdown measures and continued engagement with lockdown leisure spaces and practices (see also Clark et al. 2020).

Young people have historically been constructed as simultaneously possessing and posing ‘risk’, with their leisure practices posing a long-standing concern (Blackman and Rogers 2017). Public, semi-public and private/domestic youth leisure spaces such as street corners, nightclubs and bedrooms are key sociospatial sites where young people produce subjectivities (Roberts 2011), make connections (Abbott-Chapman and Robertson 2015) and experience intoxication (Ander and Wilińska 2020; Robinson 2009). The use of unsupervised and informal public spaces by young people—especially what Auge (1995) called non-places or temporary border zones such as doorways and staircases—has historically produced anxieties and been framed as undesirable, contentious and antisocial. They are often the focus of regulation, notably when associated with ‘risky practices’ such as substance use (Blackman 2011; Blackman and Rogers 2017; Brown 2013; Pearson 1983). It is worth remembering that (drug) policy enactment is used to practice social control on young adults in the spaces they inhabit (Gabriel et al. 2021). The liminal leisure spaces of young British-Somali men in which illicit Khat-chewing takes place are the focus of Swaine et al.’s (2018) ethnographic study^1^. Drawing on theories of social spatialisation which position spaces as sites or zones with values, representations and meanings (Shields 1991), Swaine et al. (2018) note how the young men occupied leisure spaces/times (a public stairwell is mentioned) which—being ‘betwixt and between’ (Turner 1995:95, Turner 1974) or hidden/visible—enmeshed both backstage and frontstage leisure practices, amounting to ‘the expression of secret activities in communal settings’ (Swaine et al. 2018:444) or what we might characterise as ‘private-in-public’. This draws on Erving Goffman’s concept of ‘the outside’, a third residual region or liminal space which acts both as semi-public front and semi-public backstage (Goffman 1959). As COVID-19 rules proliferated through the UK’s Coronavirus Act (2020), related statutory instruments (Hansard Society 2020) and emergency powers (Greene 2020), young people’s leisure practices, in particular the use of public, private-in-public and domestic/private spaces, drew pejorative discourse, moving beyond the illicit and undesirable towards the illegal and ‘Covidiotic’. Indeed, young people have been vilified and stigmatised as reckless pleasure seekers and rule breaking disease spreaders, who are irrational and negligent of their potential threat to wider public health (BBC News 2020; Reicher 2020). We now turn to data which suggests that this vilification and criminalisation has fallen most heavily on the shoulders of our most intersectionally disadvantaged young people experiencing enduring leisure exclusions in COVID-19 times, mirroring pre-pandemic trends.

## ‘COVID Secure’? Enduring Differentiated Youth Leisure Exclusions

The UK’s national and regional lockdowns and their proactive policing proliferated experiences of being ‘stuck at home’ with few legitimate accessible social spaces and activities (Adey et al. 2021). The liminal leisure status which intersectionally disadvantaged young people such as those in Woodrow’s study experienced pre-COVID-19 through social, cultural and geographical exclusions was experienced by young people more generally. However, more affluent young people were better placed to navigate lockdown rules and ‘safely’ socialise through technologies or in private houses/gardens, whilst digital inequalities and housing precarity resulted in disadvantaged young people being largely unable to socialise in this way (Rosenthal et al. 2020). The COVID-19 pandemic has exposed the extent of inequalities around access to inside, outside and virtual spaces of youth leisure. Inequalities in both access to and use of technology contribute to the digital exclusion of disadvantaged young people which inhibits the social benefits of online participation (Honeyman et al. 2020). Disadvantages intersect with wider crises, perpetuating inequalities through inhibited sociotechnical participation. Digital technologies have long been mooted as an inclusion panacea for young people seen as being part of a ‘digital generation’ (Buckingham and Willett 2013). However, this position fails to recognise that digital-leisure engagement can exclude as effectively as it includes. In COVID-19 times, young people experiencing intersectional disadvantages are vulnerable to digital-leisure exclusions, with virtual alternatives such as online party spaces and meetings only available to those on the ‘right’ side of the digital divide. Indeed, whilst digital spaces and virtual play saw increased participation during lockdowns (Lashua et al. 2020), the most disadvantaged young people are still not afforded the unrestricted ability to access digital spaces for socialisation.

The stop-start partial-reopening of the UK hospitality industry saw restrictions implemented to make leisure participation ‘COVID-secure’ (Cabinet Office 2020). This spawned a number of licensed socially distanced parties, such as Social Avenue in Manchester, where attendees were invited to ‘Come and dance at a distance, TOGETHER’*.* Such events provided leisure opportunities for young people who had the capital required to purchase event tickets, whilst limiting and excluding many disadvantaged young people. This highlights a prominent dimension of inclusion and exclusion from leisure activities and space being orientated around socioeconomic characteristics, evident in Woodrow’s pre-COVID-19 study (see also Batchelor et al. 2017), with financial resources allowing participation in ‘good’ legal, commercial recreational activities and spaces that more disadvantaged young people are excluded from. For Woodrow’s young people living in pre-COVID-19 times, there were a variety of inexpensive and often free activities available and engaged with such as hanging out in the park/on the streets. However, youth participation in these liminal leisure spaces during COVID-19 lockdowns was prohibited and vigorously policed. As the primary means of young people’s participation in partying moved to virtual and/or expensive COVID-secure physical spaces, the exclusion of the most disadvantaged was perpetuated, with leisure liminality emergent through intersecting disadvantages and the differential impacts of lockdown restrictions.

## Substance Use, Liminal Leisure Spaces and Policing

On reflection, the COVID-19 pandemic and intersecting crises are highlighting precisely what intersectional disadvantage entails in terms of leisure engagement: differentiated youth leisure exclusions. The following quotes from Woodrow’s pre-pandemic study highlight how the socioeconomic positions of intersectionally disadvantaged young people enable and constrain leisure practices in complex ways:

*You’re at that certain age aren’t you where you want to do stuff but you can’t, either you’re too young or too skint.* (Frank aged 21)

*Like we sit outside bus station, which looks sad, but that’s like our park, do you know what I mean? Where else is there to go?* (Ben aged 19)

In the pre-pandemic study, ‘free time’ did not necessarily equate to freedom to engage in unrestricted leisure. Here, leisure practices were bound by intersectional disadvantages of age and economic inequality, resulting in the adoption of ‘alternative’ leisure practices in liminal spaces. Similarly, throughout the pandemic, we have seen an increase in leisure time for many young people, but a reduction in leisure opportunities more generally (Roberts 2020). The young people in Woodrow’s study engaged with their liminal spaces, ascribing them with meaning, and claiming evening/night-time ownership of (ostensibly) public space through their participation and ‘place making’ practices such as collective music consumption and playing sports. There was an acute understanding of how the use of such spaces and practices had associated negative perceptions and implications. Despite this, young people still engaged in such leisure spaces, often actively defending their leisure practices:

*We’re just normal, we don’t do anything daft, but because we’re on the streets they make out we’re all criminals and druggies trying to cause trouble*. (Cameron aged 19)

For Woodrow’s sample, the leisure practices emergent in such spaces engaged friendship groups and provided valued informal social support, helping to ameliorate the pressures, stresses and anxieties of their lives (see also MacDonald and Shildrick 2007; Robinson 2009):

*It’s just same shit, different day, so you just have some weed with your mates, and it’s just nice to forget about it for a while*. (Barry aged 18)

*We just hang about around park, that’s all we do, chilling and that. If you’re with your mates having a laugh you’re not thinking about stuff*. (Oliver aged 18)

Public space for intersectionally disadvantaged young people is a key—and sometimes even the only—source of ‘private’ leisure space. Indeed, the loss of this space due to lockdown rules meant the loss of private-in-public leisure spaces for many, alongside the benefits they provide. For the young people in Woodrow’s study, leisure time was focused on street-based socialising, with substance use being an accepted and pleasurable part of their leisure activities (see also O’Gorman 2016):

*We just hang about, there’s nothing to do, let’s get stoned and have a laugh, all your mates are there, let’s have a laugh*. (Anthony aged 18)

Substance use in public spaces such as the streets was discussed by pre-pandemic study participants as a pleasurable activity, but one associated with specific ‘risks’ (see also Batchelor et al. 2017). Such risks included social embarrassment from erroneous use, and police surveillance and ‘hassle’ such as having substances confiscated, being ‘moved-on’ or sent home. They were not framed in terms of potential health harms or long-term implications from convictions:

*It doesn’t look good does it, like when you see people drinking in the park now you just think “what are you doing with yourself”, and bobbies come and you get it took off you so there’s no point.* (Todd aged 19)

*All they [police] do it take if off you, if you’re acting like an idiot, but they don’t bother with us because they know we’re not going to do anything stupid.* (Anthony aged 18)

Further, potential risk and harms ‘in the future’ such as criminal records were not a prominent feature of their appraisals. Instead, previous and potential ‘hassle’ from the police shaped leisure practices, resulting in the use of more ‘hidden’ public space—or private-in-public spaces—for their leisure practices, away from potential surveillance. This is understandable given that substance use in public spaces is widely subjected to formal and informal control mechanisms (Selfridge et al. 2020). Indeed, due to various intersecting disadvantages, the young people in the pre-pandemic study were not able to retreat into private houses and virtual spaces for leisure when faced with surveillance, regulation and social control measures. Instead, they sought out and managed their use of available leisure spaces with private-in-public potential, negotiating the ‘risks’ that accompanied these:

*You don’t want to be buzzing off your tits in the bus station do you…better off at a house party, but if there’s nothing on, nothing for us to go to, you’re just like fuck it*. (Oliver aged 18)

Enduring risks and harms from proactive policing and drug law enforcement, newly combined with coronavirus laws and rules, must be managed and negotiated by young people, notably by those already intersectionally disadvantaged. In COVID-19 times, Fixed Penalty Notices (FPN) against people breaking lockdown rules in Scotland have been issued disproportionately in the most deprived communities, with people in the ten most deprived communities being up to 12 times more likely to be issued an FPN (McVie 2020). Clear gender intersections were also evident, with men being three times more likely than women to be issued a FPN for breaking lockdown rules (McVie 2020). Indeed, data obtained from a Freedom of Information Request to the National Police Chief’s Council (NPCC) on FPN across England and Wales from 26th March 2020 to 1st January 2021 (NPCC 2021: FOI request) showed the majority of FPN (72%) being issued to men. Further, data for England and Wales between March 2020 and January 2021 showing 43% of FPN were issued to people aged 18–24 (NPCC 2021: FOI request). This shows an increase in the proportion of FPN issued to young people from earlier data between March 2020 and April 2020 when 36% of FPN were issued to people aged 18–24 (NPCC 2020). Mirroring drug law enforcement inequities (Shiner et al. 2018), data for FPN for England and Wales between March and May 2020 show Black, Asian and minority ethnic (BAME) groups being disproportionately affected, with FPN rates for BAME groups being 1.6 times higher than for White groups (Currenti and Flatley 2020). Further, BAME young men (aged 18–34) were twice as likely than White young men to be issued a FPN (ibid). Highly ‘visible’ groups of disadvantaged young people ‘hanging out’ in non-NTE spaces, as those in the pre-pandemic study did, are typically subject to police scrutiny disproportionate to their criminal or anti-social activity. Subject to increased attention from authorities and laws prohibiting assembly, intersectionally disadvantaged young people remain especially vulnerable to the criminalisation and stigmatisation of social interaction emergent during the COVID-19 pandemic.

COVID-19 has heralded continuity through the familiar presentation of disadvantaged youth as ‘problem population’ alongside changes in the ‘risk’ environments of some young people. Socialisation in public and private spaces is subject to intense public and police scrutiny and increased police targeting and harassment of youth (Selfridge et al. 2020). State intervention in the lives of young people to ‘control’ their practices within the leisure spaces they create and attend is nothing new. Classic UK studies have demonstrated that policing serves as a means of exercising social control over undesirable youth (Pearson 1983; Morris 2002). Recent attempts to regulate UK young people’s leisure practices through hefty fines (BBC News 2020, 2021), and increases in the proportion of FPN being issued to young people as outlined above, highlight continued attempts at social control of youth ‘at leisure’. Criminological scholars have critiqued the un-reflexive use of FPN based on new coronavirus legislation (Grace 2020). Disproportionate use of FPN upon young people highlights the virulent targeting of police practices. Intersectional disadvantages are being compounded by profound disruptions to already precarious youth cultures and associated leisure spaces. The COVID-19 crisis is accelerating the narrowing of young people’s ‘safe’ leisure opportunities through the exacerbation of existing differentiated leisure exclusions and the enduring proactive policing of youth sociability and intoxication practices (Measham and Moore 2008).

## Challenges and Implications of Research and Youth Work in Young People’s Leisure Spaces

Research with young people in their leisure spaces is crucial, as it allows rich, nuanced experiences and localised variations in practices to be captured. Young people in Woodrow’s pre-COVID-19 study were accessed through attending established in-person outreach youth work, which sought to engage young people not typically in contact with services in their public and public-in-private leisure spaces and times. Access was enabled through Woodrow’s partial insider status as an outreach worker in the data collection site, and experience growing up in the local area. Whilst the research would not have been impossible to complete without this access, it would have been difficult and time-consuming to establish presence and trust without these structures and experiences being in place. The young people in the pre-COVID-19 study were forced into more ‘hidden’ public spaces and, when possible, into private/domestic spaces due to their illicit leisure practices, making research and access challenging. From our experiences of working in youth and drug outreach, this raises concerns around youth substance use practices and the negotiation of ‘risks’ in the rapidly changing leisure landscape of COVID-19 times and beyond. The impacts of COVID-19 upon intersectionally disadvantaged young people’s leisure through lockdown rules and police scrutiny alongside young people moving into more hidden and ‘hard to reach’ physical and virtual spaces mean that access to young people to understand their experiences becomes more challenging. Having leisure spaces limited, removed, stigmatised and criminalised has a profound effect on young people’s mental health and wellbeing (Roberts 2020; NHS Digital 2020). This impact upon young people’s long-term wellbeing is a pressing concern for youth studies and youth work. However, youth research and youth work on the impact of the pandemic on young people has been curtailed by measures meant to manage the risk of COVID-19 to the UK wider population, such as minimising in-person interactions, as experienced during Woodrow’s recent research with young people in the Public Health field. This may be described as the ‘covidisation’ of youth research, where the ‘risks’ young people are thought to present to others are assumed to be in greater need of mitigation than the risks of not doing research and youth work with them. Intersectionally disadvantaged young people ‘at leisure’ are then subject to a particularly problematic confluence of criminalisation, exclusion and stigmatisation in COVID-19 times, a pernicious trend to be countered by youth researchers, youth workers and young people themselves.

## Conclusion

The position and positioning of young people in COVID-19 times combines strains of continuity and change. Foregrounding continuity and change means not exploring everything solely through the lens of COVID-19, however tempting that may be in ‘unprecedented times’. In exploring the continuities as well as changes in youth leisure experiences, differentiated according to intersectional disadvantages apparent prior to and during the pandemic, we hope to have highlighted opportunities to use insights from pre-pandemic studies to understand the contemporary context. This includes the interdisciplinary intersections of youth studies and criminology which pay attention to the policing of those young people inhabiting liminal leisure spaces who are largely excluded from legal commercialised leisure spaces. Leisure liminality is exacerbated by intersectional disadvantages, compounded by disruptions to youth leisure spaces and practices given the COVID-19 pandemic and the UK government responses to it. The COVID-19 pandemic has worsened intersectional disadvantages in youth leisure, notably the differential availability or otherwise of public, private-in-public, private/domestic and virtual spaces. Lockdown restrictions have meant that the leisure liminality of disadvantaged young people pre-pandemic through economic, sociocultural and geographical exclusions temporarily emerged as the experience of young people more generally. However, whilst all young people must manage their desire for pleasure and leisure in a context of social distancing, digital divides, rising NTE participation costs and proactive policing, some remain better placed than others to negotiate ‘risks’ and potential harms from criminalisation for example. Indeed, young people experiencing intersectional disadvantages are especially susceptible to differentiated leisure exclusions and the criminalisation of social interaction in COVID-19 times and beyond. Drawing on pre-COVID-19 work with intersectionally disadvantaged young people, we note how when subject to increased policing and social control measures illicit leisure practices were not abandoned but moved to more liminal leisure spaces, with implications for connecting with such young people. The continuation of existing leisure exclusions and the emergence of novel forms in youth leisure landscapes post-pandemic presents a unique challenge to those researching and working with intersectionally disadvantaged young people for whom leisure—‘chilling and that’—remains essential.

## Data Availability

Data are available upon reasonable request.

## Footnotes

1 Khat (Catha edulis) is a shrub-like narcotic consumed for recreational purposes as a mild stimulant by cultures primarily located in the Horn of Africa and the Arabian Peninsula. In the UK, it is predominately consumed by British Somali men, although its use among young men is culturally prohibited (Swaine et al. 2018). In 2014 Khat was classified as a Class C substance under the Misuse of Drugs Act 1971, prompting concerns among drug policy change campaigners about the criminalisation of a specific population.
